# Transcriptional and epigenetic control of human naïve CD8^+^ T cell activation

**DOI:** 10.3389/fimmu.2026.1749526

**Published:** 2026-01-27

**Authors:** Yaqiu Hu, Yuxia Du, Xuenuo Chen, Yongguo Li, Cheng Yang

**Affiliations:** Chongqing Key Laboratory of Infectious Diseases and Parasitic Diseases, Department of Infectious Diseases, The First Affiliated Hospital of Chongqing Medical University, Chongqing, China

**Keywords:** naïve CD8^+^ T cell activation, epigenetic dynamics, functional differentiation, metabolic reprogramming, transcriptional profiles

## Abstract

**Background:**

The differentiation of naïve CD8^+^ T cells into effector cells upon activation is essential for eliminating intracellular pathogens and cancerous cells, although the underlying epigenetic mechanisms remain incompletely characterized.

**Methods:**

Peripheral blood mononuclear cells (PBMCs) were obtained from healthy donors. naïve CD8^+^ T cells were purified and activated with α-CD3/CD28-conjugated microbeads for 0, 24, or 72 h *in vitro*. Flow cytometry was used to assess cytokine production and activation markers at each time point. Assay for transposase-accessible chromatin using sequencing (ATAC-seq) was performed to identify differentially accessible chromatin regions (DARs). RNA sequencing (RNA-seq) was performed to measure gene expression. Data from ATAC-seq and RNA-seq were integrated to examine the relationship between chromatin accessibility and gene expression. Enriched pathways for DARs and differentially expressed genes (DEGs) were determined by KEGG pathway and gene ontology (GO) enrichment analysis, and transcription factor (TF) binding patterns around these genes were visualized by footprint analysis.

**Results:**

Upon activation, naïve CD8^+^ T cells showed increased production of IFN-γ, TNF, and IL-2, and elevated expression of CD69 and CD95. Integrated ATAC-seq and RNA-seq analysis identified 568 and 541 dual-upregulated genes (showing both increased chromatin accessibility and expression) at 24 and 72 h post-activation, respectively. These early-response genes were enriched in pathways including pyruvate metabolism and the DNA damage response. Footprint analysis predicted the ETS and bZIP TF families as key regulators driving this coordinated chromatin and transcriptional reprogramming. Furthermore, distinct chromatin remodeling patterns were observed in gene sets associated with memory, effector function, exhaustion, and metabolism, revealing that accessibility changes did not always directly correlate with transcriptional outcomes.

**Conclusion:**

This study defines a core set of genes and TFs that critically regulate the initial activation of human naïve CD8^+^ T cells. These results provide a molecular roadmap for future efforts to engineer more potent and durable CD8^+^ T cell responses for adoptive cell therapy.

## 1 Introduction

Effector CD8^+^ T cells (T_E_) play a crucial role in eliminating intracellular pathogens and tumor cells through multiple mechanisms including: 1) secretion of cytokines such as IFN-γ, TNF, and IL-2 (1); 2) release of effector molecules including perforins and granzymes (2, 3); and 3) FAS-FASL-mediated interactions with target cells (4, 5). CD8^+^ T_E_ cells are generated from naïve T cells (T_N_) following optimal priming with TCR and co-stimulatory signals (6), or from memory T cells (T_M_) upon antigen re-encounter. While the generation of a large T_E_ pool from T_N_ cells typically requires several days of clonal expansion, T_M_ cells can rapidly differentiate into T_E_ cells with greater magnitude and faster kinetics (7, 8). Consequently, the mechanisms underlying the rapid responsiveness of T_M_ cells have been extensively investigated (9, 10). In contrast, the early activation signatures of T_N_ cells, which are essential for primary infection clearance and the establishment of memory populations, remain less comprehensively understood.

In eukaryotic cells, DNA accessibility for gene transcription is governed by chromatin structure. Epigenetic modifications—including DNA methylation and histone post-translational modifications (hPTMs)—play critical roles in regulating CD8^+^ T cell differentiation and function (11, 12). For example, functional memory CD8^+^ T cells acquire elevated histone acetylation at effector gene loci such as *IFNG*, *PRF1*, and *GZMB*, enabling rapid transcriptional reactivation upon re-stimulation (13, 14). Genome-wide profiling of DNA methylation and hPTMs has further delineated the epigenetic landscapes of effector (T_E_) and memory (T_M_) cell identities (15, 16). Dynamic histone modifications, such as loss of H3K27me3 and gain of H3K4me3, have also been linked to transcriptional reprogramming during effector differentiation (17, 18).

Since epigenetic states strongly influence chromatin accessibility and thus transcription factor binding, the development of ATAC-seq has transformed this field by enabling genome-wide mapping of open chromatin regions (19). Indeed, studies using ATAC-seq have confirmed that naïve, effector, memory, and exhausted T cell subsets possess distinct and stable chromatin accessibility profiles (15, 20). Notably, epigenetic reprogramming that drives T cell differentiation begins rapidly after activation: in mouse models, observable changes occur within days of antigen exposure (21), while single-cell analyses reveal transcriptional heterogeneity as early as the first cell division, supporting an early bifurcation model of differentiation (22). Multi-omics studies in infection models have further identified key transcription factors that regulate T cell subset fate (23).

However, most of these insights derive from animal models (20, 24) and often focus on later time points. Although recent atlases have begun to map chromatin landscapes in human immune cells (25), a systematic and dynamic analysis of the earliest stages of human naïve CD8^+^ T cell activation—particularly the interplay between chromatin remodeling and transcriptional output within hours post-stimulation—remains lacking.

To address this critical gap, we performed an integrated ATAC-seq and RNA-seq analysis to dynamically profile the chromatin accessibility and transcriptional landscapes of human naïve CD8^+^ T cells during early *in vitro* activation (24 and 72 h). Our study captures the dynamic changes in gene expression and chromatin accessibility within this critical initial window. Moreover, we identified enriched TF binding motifs associated with early activation, providing mechanistic insights into the initiating events of T cell activation. Collectively, our work unveils the early epigenetic and transcriptional events in human naïve CD8^+^ T cell activation, offering a novel and fundamental resource for understanding T cell biology and informing the rational design of T-cell-based therapies.

## 2 Materials and methods

### 2.1 Isolation and activation of human naïve CD8^+^ T cells *in vitro*

PBMCs were isolated from healthy donors using Ficoll-Paque™ PREMIUM density gradient centrifugation (Cytiva, Cat. #17544203) (26, 27). naïve CD8^+^ T cells were then enriched through negative selection using EasySep™ Human Naïve CD8^+^ T Cell Isolation Kit (STEMCELL, Cat. #19258) according to the manufacturer’s protocol, achieving a purity of 90–95% as routinely verified. The enriched naïve CD8^+^ T cells were resuspended in RPMI-1640 medium with 10% FBS and seeded in a 96-well plate at a density of 2×10^6^ cells/mL. The cells were immediately stimulated with Dynabeads™ Human T- Activator CD3/CD28 (Gibco, Cat. #11131D) at a cell to bead ratio of 1:1 for 0, 24, or 72 h. At each specified time point, cells were harvested for subsequent analysis by flow cytometry, RNA-seq, and ATAC-seq. This study was approved by the Ethics Committee of the First Affiliated Hospital of Chongqing Medical University (approved date: 2020-12-14; approval number: 2020-731). Informed consent was obtained from all volunteers, and all experiments were conducted in accordance with the Declaration of Helsinki. All procedures were performed in compliance with relevant laws and institutional guidelines.

### 2.2 Flow cytometry

Protocols for antibody staining have been described previously (28). Briefly, cells were initially incubated with LIVE/DEAD™ Fixable Aqua Dye (Invitrogen, Cat. #L34989), then stained with the following antibodies against surface markers for 15 minutes at 4°C: anti-CD3-BV421 (BioLegend, clone UCTH1, Cat. #300434), anti-CD8-BV650 (BioLegend, clone RPA-T8, Cat. #301042), anti-CD45RA-PerCP-Cy5.5 (BioLegend, clone HI100, Cat. #304122), anti-CD27-APC-Cy7 (BioLegend, clone O323, Cat. #302816), anti-CD69-APC (BioLegend, clone FN50, Cat. #310910), anti-CD122-BV786 (BD Biosciences, clone Mik-β3, Cat. #743118), and anti-CD95-PE (BioLegend, clone DX2, Cat. #305608). For detection of IFN-γ, TNF, and IL-2 production by intracellular cytokine staining (ICS), cells were stimulated as previously described. Six hours prior to the end of stimulation, GolgiStop (BD Biosciences, Cat. #554724) was added at a 1:1000 dilution. After stimulation, cells were stained with viability dye and surface antibodies as described above; fixed and permeabilized using Cytofix/Cytoperm solution (BD Biosciences, Cat. #554714); and then stained with the following antibodies specific to cytokines: anti-IFN-γ-APC (BioLegend, clone B27, Cat. #506510), anti-TNF-PE-CF594 (BioLegend, clone MAb11, Cat. #502946), and anti-IL-2-AF700 (BioLegend, clone MQ1-17H12, Cat. #500320). After staining, cells were resuspended in FACS buffer for acquisition using a FACS Fortessa flow cytometer (BD Biosciences). Data were analyzed using FlowJo (v. 10.9.0) software (Treestar).

### 2.3 RNA-seq

Total RNA was extracted using TRIzol (Invitrogen, Cat. #15596026CN). The quality of RNA was determined using Qubit RNA Broad Range Assay Kit (Life Technologies, Cat. #Q10210). cDNA library preparation and sequencing were performed by Wuhan Seqhealth Company (Wuhan, China) following standard protocols. Procedures for RNA-seq data analysis have been described previously (29). In brief, adapter and low-quality reads were filtered using Trimmomatic (v. 0.36), followed by duplicates removal with UMI soft in-house (v. 1.0). Mapping of reads to the genome was conducted with STAR (v. 2.5.3a). Calculation of gene expression was performed using featureCounts (v. 1.5.1) and edgeR (v. 3.12.1) software. DEGs were identified using DESeq2 with the following criteria: |log_2_ fold change| > 1 and adjusted *P*-value < 0.05.

### 2.4 ATAC-seq

#### 2.4.1 Transposition assay, library preparation and sequencing

ATAC-seq sample preparation was performed as previously described (19). Briefly, 10,000–50,000 cells were resuspended in 50 μL of cold lysis buffer, nuclei were pelleted by centrifugation (500 g, 5 min, 4 °C), resuspended in 50 μL of transposition reaction mix, and incubated at 37 °C for 30 min. High-throughput DNA sequencing library preparation was carried out using TruePrep DNA Library Prep Kit (Vazyme, Cat. #TD501). The final library products were amplified, quantified, and sequenced on an Illumina Novaseq 6000 platform with a 150-bp paired-end configuration.

#### 2.4.2 Bioinformatic analysis

Low-quality reads were discarded and reads contaminated with adaptor sequences were trimmed using fastp (v. 0.23.1). The resulting clean reads were mapped to the human reference genome GRCh38/hg38 using bowtie2 (v. 2.2.6) with default parameters. The distribution of mapped reads, coverage uniformity, and strand specificity was evaluated using RSeQC (v. 2.6). The fragment size distribution was determined using the Collect Insert Size Metrics tool from Picard (v. 2.8.2). The enrichment signal around transcription start site (TSS) was visualized with DeepTools (v. 2.4.1). Genome-wide regions of open chromatin (peaks) were performed for each sample individually using MACS2 (v. 2.1.1). The resulting peaks were annotated, and their genomic distribution was analyzed using BEDTools (v. 2.30.0).

To identify differentially accessible peaks (DAPs) between comparison groups, a consensus set of peaks was first created by merging all identified peaks across all samples. Read counts for each peak in the consensus set were quantified for every sample. Differential analysis was performed using the csaw package (v. 1.24.3) within the edgeR framework in R. Peaks with an adjusted *P*-value (FDR) < 0.05 and an |log2 fold change| > 1 were defined as statistically significant DAPs. *De novo* motif discovery and enrichment analysis within DAPs were performed using HOMER (v. 4.10).

### 2.5 GO and KEGG pathway enrichment analysis, motif enrichment and footprint identification

Gene Ontology (GO) analysis and Kyoto Encyclopedia of Genes and Genomes (KEGG) enrichment analysis for DEGs were conducted using KOBAS software (v. 2.1.1), with a *P*-value cutoff of 0.05 to determine statistically significant enrichment. Microsoft Excel 2013 was used for data extraction, sorting, and cleaning, while statistical analyses were conducted using R (v. 4.2.2), Stata (v. 17.0), and Joinpoint (v. 4.9.0.0).

### 2.6 Statistical analysis

Statistical analysis was performed on the levels of intracellular cytokine secretion, surface marker expression, and differentially expressed genes across the three time points. Data are presented as median with interquartile range. Due to the small sample size (n=4) and the data failing the Shapiro-Wilk test for normality, nonparametric tests were employed for between-group comparisons. The Friedman test was first used to assess whether an overall statistically significant difference existed among the three time points. If the Friedman test indicated a significant overall difference (*P* < 0.05), pairwise comparisons were subsequently conducted using Dunn’s multiple comparisons test. All analyses were performed using GraphPad Prism (v. 10.0).

## 3 Results

### 3.1 Phenotypic, transcriptional, and chromatin accessibility profiles during human naïve CD8^+^ T cell activation

**Figure 1:**
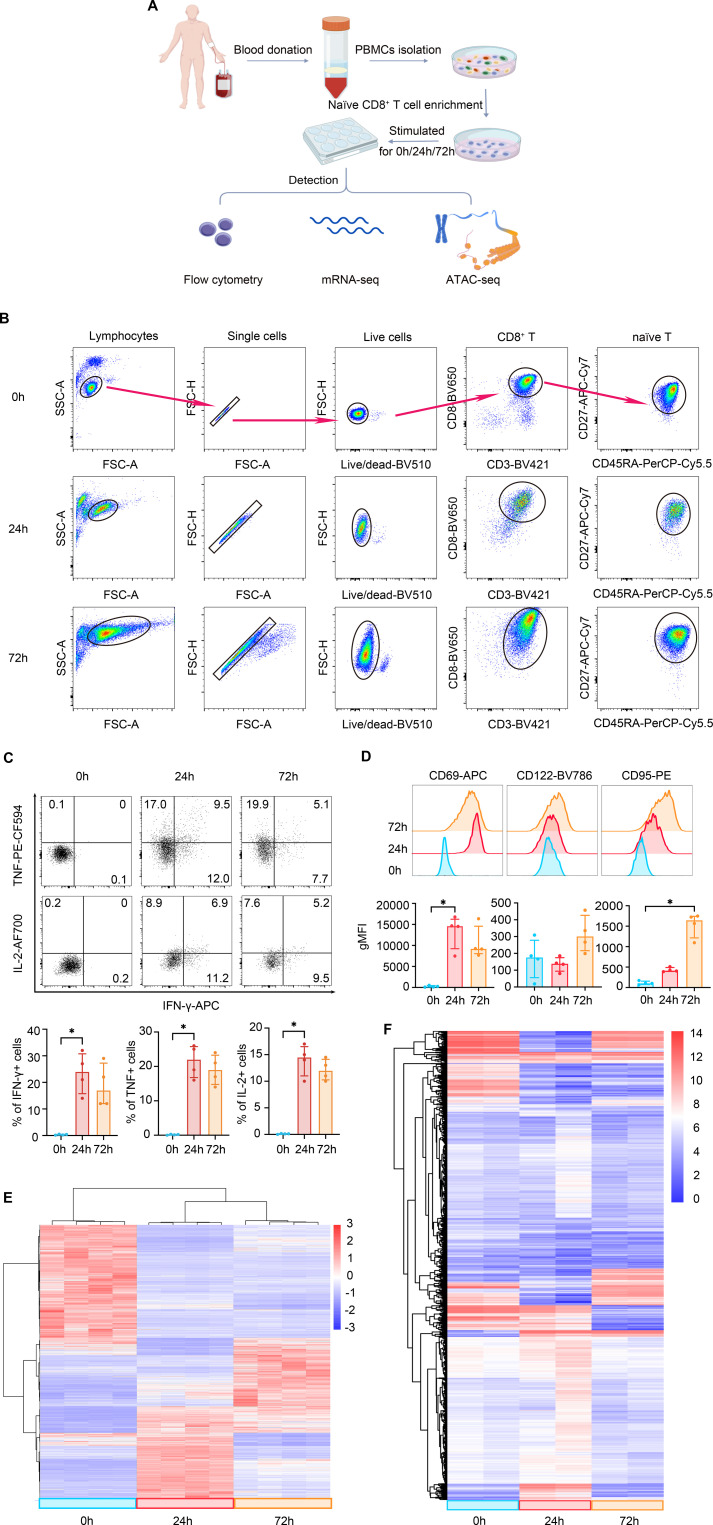
Phenotypic, transcriptional, and chromatin accessibility profiles during naïve CD8^+^ T cell activation. **(A)** Experimental design and analysis workflow. **(B)** Flow cytometry analysis of naïve CD8^+^ T cell size and viability at indicated time points post-stimulation. **(C)** Intracellular cytokine staining depicting IFN-γ, TNF, and IL-2 production in naïve CD8^+^ T cells at 0, 24, and 72 h post-activation. **(D)** Expression of activation markers CD69, CD122, and CD95 in naïve CD8^+^ T cells over time. Top, representative histograms; bottom, summary of geometric mean fluorescence intensity (gMFI). **(E)** Heatmap of differentially expressed genes (DEGs), with hierarchical clustering identifying three distinct states: quiescent (0 h), early activation (24 h), and effector expansion (72 h). **(F)** Heatmap of chromatin accessibility profiles from ATAC-seq, demonstrating global remodeling synchronized with the transcriptional changes shown in **(E)**.

Although previous studies have shown that the epigenetic modifications landscapes are distinct among CD8^+^ T cell subsets (30), it remains unclear whether epigenetic remodeling occurs in naïve CD8^+^ T cells during the early stages of activation. To address this, we stimulated purified naïve CD8^+^ T cells with anti-CD3/CD28 up to 72 h, and compared the phenotypic, transcriptional, and epigenetic changes at 24 and 72 h post stimulation, compared to non-stimulated cells (0 h) (**Figure 1A**). Phenotypically, naïve CD8^+^ T cells exhibited a gradual increase in size following anti-CD3/CD28 stimulation, with nearly all cells remaining viable after 72 h (**Figure 1B**). This stimulation also induced a robust effector response. The percentages of cells secreting IFN-γ, TNF, and IL-2 were significantly elevated post-stimulation (**Figure 1C**). Concurrently, the expression of activation markers, including CD69 and CD95, was upregulated, alongside a moderate increase in CD122 (**Figure 1D**).

Beyond these phenotypic changes, transcriptomic profiling revealed a precise and stepwise reprogramming of the gene expression landscape. Hierarchical clustering of differentially expressed genes (DEGs) clearly delineated three distinct temporal patterns corresponding to the quiescent (0 h), early-activation (24 h), and effector-expansion (72 h) phases, indicating that T cell activation follows a strictly regulated temporal program rather than a gradual drift (**Figure 1E**). Importantly, this transcriptional divergence was mirrored by the chromatin accessibility landscape. ATAC-seq analysis demonstrated a global reorganization of the epigenome, where genomic regulatory regions underwent synchronized opening or closing to support the specific transcriptional demands of each activation stage (**Figure 1F**). Collectively, these data demonstrate that the phenotypic activation of naïve CD8^+^ T cells is driven by a robust and synchronized orchestration of epigenetic and transcriptional remodeling, establishing distinct molecular states at early and late activation time points.

### 3.2 Enriched biological processes of differentially regulated genes in naïve CD8^+^ T cells following activation

**Figure 2:**
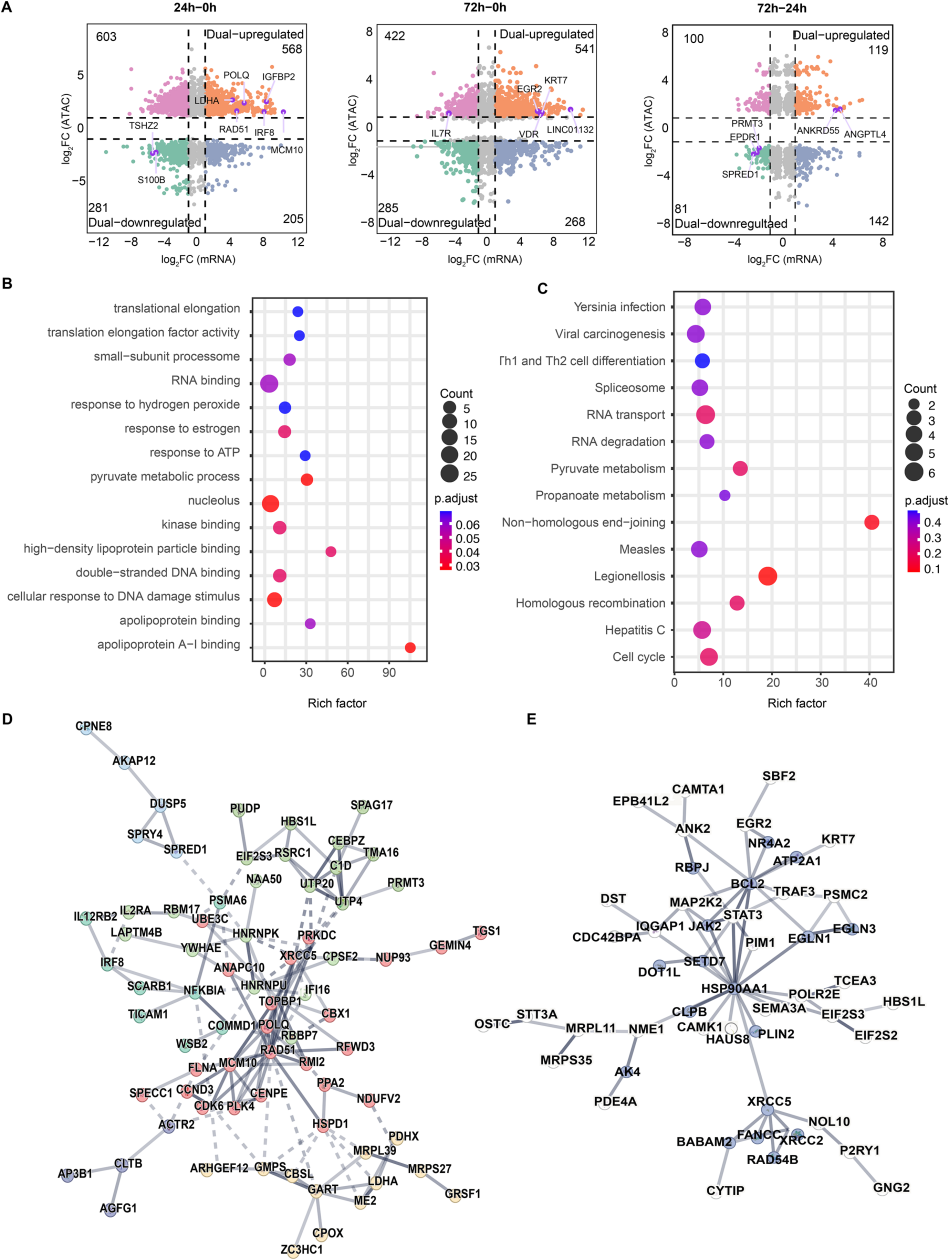
Enriched biological processes of differentially regulated genes in naïve CD8^+^ T cells during activation. **(A)** Integrated ATAC-seq and RNA-seq analysis showing upregulated genes in more accessible chromatin regions (“dual-upregulated” genes, orange) and downregulated genes in less accessible regions (“dual-downregulated” genes, green). Thresholds: |log_2_(fold change)| > 1, *P* < 0.05. **(B)** GO enrichment analysis of dual-upregulated genes (24 h vs. 0 h). **(C)** KEGG pathway enrichment analysis of dual-upregulated genes (24 h vs. 0 h). **(D)** Protein-protein interaction (PPI) network of dual-upregulated genes (24 h vs. 0 h), displaying only interacting genes with functional clusters represented by distinct colors. **(E)** PPI network of dual-upregulated genes (72 h vs. 0 h), with stress response-related genes highlighted in purple.

To investigate the correlation between gene expression and chromatin accessibility, we performed an integrated analysis of ATAC-seq and RNA-seq data. Compared to non-activated cells (0 h), 568 genes exhibited increased chromatin accessibility and robust expression (dual-upregulated) in cells that were stimulated with anti-CD3/CD28 for 24 h, while 281 genes showed reduced accessibility and decreased expression levels (dual-downregulated). Among these genes, *MCM10*, *IGFBP2*, *IRF8*, *POLQ*, *RAD51*, and *LDHA* were the top ranked dual-upregulated genes, whereas *S100B* and *TSHZ2* were remarkably downregulated. The number of dual-upregulated and downregulated genes between 72 h and 0 h was 541 and 285, respectively. Notably, far fewer genes were differentially regulated between 72 h and 24 h (**Figure 2A**). Additionally, some genes displayed reciprocal transcription and chromatin accessibility profiles, suggesting that gene expression is not solely determined by chromatin accessibility.

Next, enriched biological functions of differentially regulated genes were predicted by GO analysis. We first focused on genes showing consistent changes in transcription and chromatin accessibility. The results revealed that dual-upregulated genes between 24 h and 0 h were enriched in apolipoprotein A-I binding, cellular response to DNA damage stimulus, and pyruvate metabolic process (**Figure 2B**). Similarly, a significant enrichment of genes involved in pyruvate metabolism was observed by KEGG pathway analysis (**Figure 2C**). To identify potential key regulators of these significantly enriched biological processes, we constructed a protein-protein interaction (PPI) network. Within the DNA repair pathway (highlighted in red), *RAD51*, *TOPBP1*, and *MCM10* emerged as central hubs (**Figure 2D**). Consistent with this, *MCM10* is known to play a critical role in initiating DNA replication and preventing DNA damage, while *RAD51* is essential for homologous strand exchange during homologous recombination (31). Within the pyruvate metabolism module (highlighted in yellow), *GART*, *LDHA*, and *MRPL39* were identified as key network nodes (**Figure 2D**). At 72 h, key TCR downstream signaling components such as *MAP2K2*, along with transcription factors including *EGR2*, *NR4A2*, and *BCL2*, occupied central positions in the PPI network. *STAT3*, a regulator of T cell fate, was also prominent (**Figure 2E**). Together, these results suggest that biological processes such as DNA repair and pyruvate metabolism may play critical roles during early naïve CD8^+^ T cell activation, while remodeling of TCR signaling pathways is associated with effector expansion.

### 3.3 Identification of master TFs and visualization of their footprints

**Figure 3:**
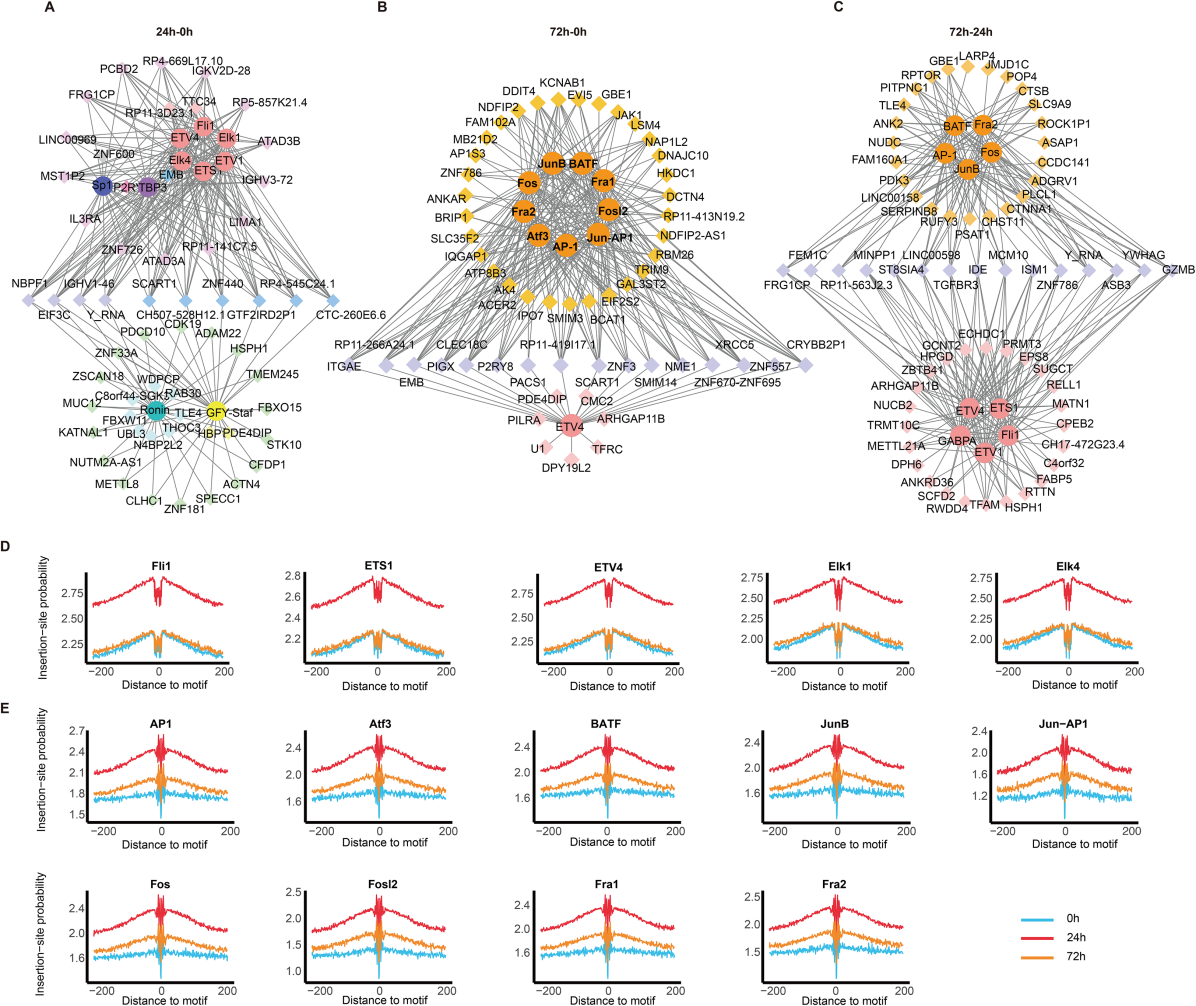
Identification of master TFs and their DNA binding footprints. **(A–C)** Regulatory networks showing interactions between the top 10 predicted TFs (based on motif analysis) and their 30 target genes for: **(A)** 24 h vs. 0 h, **(B)** 72 h vs. 0 h, and **(C)** 72 h vs. 24 h comparisons. (D & E) Footprint analysis of **(D)** ETS family and **(E)** bZIP family transcription factors at 0, 24, and 72 h time points.

Chromatin accessibility is considered to provide the foundation for TF binding, which subsequently facilitates the initiation of gene transcription. Motifs, often recognized as TF binding sites, were analyzed in the promoter regions of differentially regulated genes to identify critical TFs involved in naïve CD8^+^ T cell activation. The top ten predicted TFs within top thirty DEGs between each pair of groups are shown in **Figures 3A–C**. The results revealed that DEGs between 24 h and 0 h were predominantly enriched with motifs for Fli1, ETS1, ETV4, ETV1, Elk1, and Elk4, all of which belong to the ETS family. Most target genes of these ETS family TFs were also enriched with motifs for Sp1 and TBP3. In contrast, Ronin and GFY-Staf shared few target genes with ETS family (**Figure 3A**). The bZIP family TFs, including JunB, AP-1, Fos, Fra2, Atf3, BATF, Fosl2, Fra1, and Jun-AP1, were predicted to be major regulators of DEGs at 72 h compared to 0 h and 24 h (**Figures 3B, C**). Notably, many of these DEGs were also enriched with motifs for ETS family TFs (**Figures 3B, C**).

Next, the potential binding of TFs to different genomic regions was visualized through footprint analysis. As expected, the most probable binding sites for all TFs overlapped with the predicted motif locations, and binding probability decreasing with distance from the motif center (**Figures 3D, E**). Interestingly, footprint signals for all predicted TFs in both the ETS (**Figure 3D**) and bZIP families (**Figure 3E**) were markedly stronger at 24 h, suggesting increased binding affinity or occupancy at this time point. In summary, the ETS and bZIP family TFs may serve as critical regulators of gene expression programs during naïve CD8^+^ T cell activation.

### 3.4 Chromatin accessibility and transcription dynamics of genes associated with memory differentiation and effector function in naïve CD8^+^ T cells

**Figure 4:**
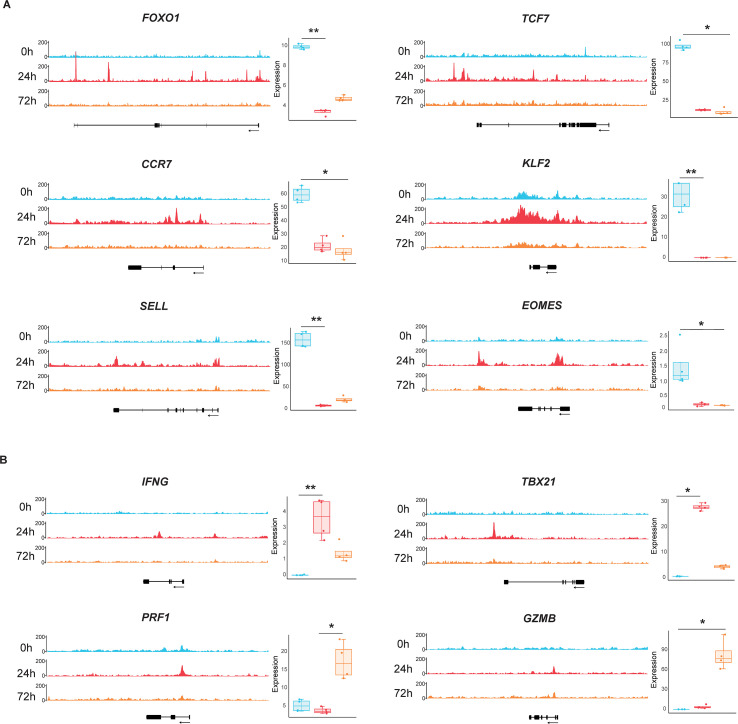
Chromatin accessibility and transcriptional dynamics of memory versus effector-associated genes in naïve CD8^+^ T cells. **(A)** Coordinated chromatin accessibility (ATAC-seq) and mRNA expression (RNA-seq) profiles for memory-associated genes: *FOXO1* (forkhead box O1), *TCF7* (transcription factor 7), *CCR7* (chemokine receptor 7), *KLF2* (Kruppel-like factor 2), *SELL* (selectin L), and *EOMES* (eomesodermin). **(B)** Corresponding chromatin accessibility and transcriptional profiles for effector-associated genes: *IFNG* (interferon gamma), *TBX21* (T-box transcription factor 21), *PRF1* (perforin 1), and *GZMB* (granzyme B).

Having comprehensively characterized the chromatin accessibility landscape and transcriptional profiles during naïve CD8^+^ T cell activation, including DEGs, enriched biological pathways, TF binding motifs, and regulatory networks, we next investigated the transcriptional and epigenetic regulation of genes critical for CD8^+^ T cell function. Specifically, we prioritized our focus on genes associated with T cell memory formation and effector differentiation. The results showed that expression levels of memory-associated genes (*FOXO1*, *TCF7*, *KLF2*, *CCR7*, *SELL*, and *EOMES*) were the highest in quiescent naïve CD8^+^ T cells, which were markedly downregulated upon activation (**Figure 4A**). Chromatin accessibility at these gene loci generally decreased during activation, although *CCR7* and *SELL* displayed transiently increased accessibility at 24 h post-stimulation. Consistent with the chromatin accessibility pattern, expression of *IFNG* and *TBX21* peaked at 24 h post-stimulation. Strikingly, *PRF1* and *GZMB* reached maximal expression at 72 h despite reduced chromatin accessibility at this late time point (**Figure 4B**). These findings indicate that chromatin accessibility correlates with expressions levels for only a subset of memory- and effector function-associated genes.

### 3.5 Chromatin accessibility dynamics and transcriptional regulation of T cell exhaustion- and metabolism-associated genes during naïve CD8^+^ T cell activation

**Figure 5:**
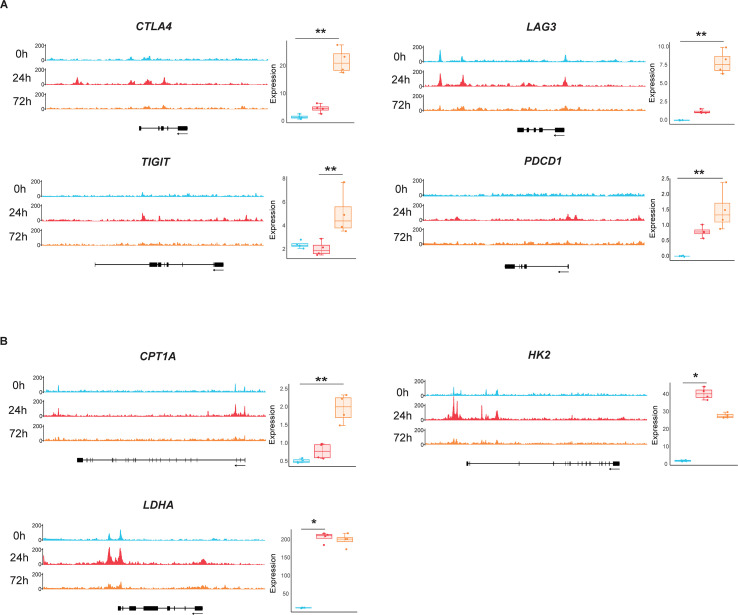
Chromatin accessible patterns and transcriptional regulation of T cell exhaustion- and metabolism-associated genes. **(A)** Integrated chromatin accessibility and transcriptional profiles of exhaustion-associated genes: *CTLA4* (cytotoxic T-lymphocyte-associated protein 4), *LAG3* (lymphocyte-activation gene 3), *TIGIT* (T cell immunoreceptor with Ig and ITIM domains), and *PDCD1* (programmed cell death protein 1). **(B)** Coordinated chromatin accessibility and mRNA expression patterns of metabolism-associated genes: *CPT1A* (carnitine palmitoyltransferase 1A), *HK2* (hexokinase 2), and *LDHA* (lactate dehydrogenase A).

Under chronic antigen stimulation, T cells develop exhaustion, a dysfunctional state characterized by progressive loss of effector functions and sustained upregulation of multiple inhibitory receptors (IRs) (32). To determine whether transcriptional and epigenetic remodeling of IR-encoding genes is initiated during early T cell activation, we assessed chromatin accessibility and transcriptional levels of these genes in naïve CD8^+^ T cells at baseline (0 h) and 24/72 h post-stimulation. The data showed that transcriptional levels of *CTLA4*, *LAG3*, *TIGIT*, and *PDCD1* were significantly upregulated by 72 h post-activation. However, this transcriptional increase was not accompanied by enhanced chromatin accessibility at their respective loci, suggesting uncoupling of epigenetic and transcriptional regulation for these genes (**Figure 5A**).

The transition from naïve to effector T cells requires metabolic reprogramming to fuel proliferation and effector molecule synthesis. Activated T cells shift from oxidative phosphorylation to aerobic glycolysis, a transition coordinated with dynamic changes in metabolic enzyme expression (33). Carnitine palmitoyl-transferase (*CPT1A*), the rate-limiting enzyme in mitochondrial fatty acid oxidation, exhibited low baseline expression in resting naïve CD8^+^ T cells, followed by progressive upregulation during activation (**Figure 5B**). In contrast, *HK2* and *LDHA*, which encode the glycolytic enzymes hexokinase 2 and lactate dehydrogenase A, were rapidly induced by 24 h post-stimulation and maintained elevated expression through 72 h (**Figure 5B**). Notably, chromatin accessibility at these metabolic gene loci peaked at 24 h post-stimulation, which did not fully correlate with transcriptional dynamics (**Figure 5B**). Together, these data indicate that early naïve CD8^+^ T cell activation triggers transcriptional upregulation of exhaustion- and metabolism-associated genes independent of chromatin accessibility remodeling.

## 4 Discussion

To elucidate epigenetic mechanisms governing naïve CD8^+^ T cell activation, we generated genome-wide chromatin accessibility landscapes (ATAC-seq) and transcriptional profiles (RNA-seq) across multiple time points (0, 24, and 72 h post-stimulation). Activated naïve CD8^+^ T cells exhibited elevated cytokine secretion (IFN-γ, TNF, and IL-2) and upregulated surface activation markers (e.g., CD69, CD95), accompanied by dynamic chromatin accessibility and transcriptional reprogramming. Key biological processes enriched during activation included DNA repair, pyruvate metabolism, and cellular stress responses (**Figures 2B, C**). TF motif analysis revealed that ETS and bZIP family members were significantly enriched among DEGs, suggesting their roles as master regulators of activation-associated transcriptional networks (**Figures 3A–C**). Notably, genes linked to memory differentiation (*TCF7*, *KLF2*), effector functions (*GZMB*, *PRF1*), inhibitory receptors (*CTLA4*, *LAG3*), and metabolic reprogramming (*HK2*, *CPT1A*) underwent both chromatin remodeling and differential expression. However, chromatin accessibility changes were only partially correlated with transcriptional dynamics, indicating context-dependent regulatory mechanisms (**Figures 4**, **5**).

Our integrated multi-omics analysis reveals the dynamic regulatory hierarchy of naïve CD8^+^ T cells transitioning from a quiescent state (0 h) to early activation (24 h) and subsequently to effector- expansion (72 h). The significant coordination between chromatin accessibility dynamics and transcriptional changes suggests that chromatin remodeling is a major, prerequisite driver of T cell fate determination. While not universal, as some gene expression changes occurred independently of accessibility shifts, this coordinated pattern underscores the foundational role of epigenetic reprogramming in establishing early activation states (25). At 24 h post-activation, T cells primarily undergo metabolic reprogramming and preparation for clonal expansion. Differential analysis (24 h vs. 0 h) shows that dual-upregulated genes (accessible chromatin and increased expression) are significantly enriched in pyruvate metabolic process and response to ATP pathways (**Figures 2B, C**). This indicates a metabolic shift from oxidative phosphorylation to aerobic glycolysis (the Warburg effect), providing the necessary carbon sources and energy for rapid proliferation (34). Consistent with this, GO analysis highlights enrichment in translational elongation and small-subunit processome, reflecting robust protein synthesis. This provides a molecular explanation for the significant increase in cell size (T cell blasting) observed in our flow cytometry and FSC/SSC scatter plots (**Figure 1B**). Crucially, PPI network analysis identified *MCM10*, *RAD51*, and *POLQ* as central hubs (**Figure 2D**). The upregulation and chromatin accessibility of *MCM10*, a key initiator of DNA replication, suggest that T cells have crossed the G1/S checkpoint and are initiating DNA unwinding (35). However, rapid division imposes substantial replication stress. The synchronous upregulation of *RAD51* (homologous recombination repair) and *POLQ* (DNA polymerase), along with the enrichment of the homologous recombination pathway in KEGG analysis, indicates that T cells establish stringent genome protection mechanisms to prevent replication fork collapse and DNA damage during burst proliferation (36). Additionally, the early epigenetic opening of the transcription factor *IRF8* likely facilitates the transcriptional programming of early inflammatory responses (37).

At 72 h, T cells transition into a phase of signal maintenance and effector function execution. Although IL-2 secretion is detected as early as 24 h, PPI analysis (**Figure 2E**) reveals that the *STAT3*, the downstream transcription factor of IL-2 receptor subunit IL2RA (CD25), remains at the regulatory core at 72 h. This suggests that sustained IL-2/STAT3 signaling is indispensable for maintaining T cell viability and effector status during this phase (38). Concurrently, the PPI network features TCR downstream signaling molecules (e.g., *MAP2K2*) alongside regulators, such as *EGR2*, *NR4A2*, and *BCL2*. While *EGR2* and *NR4A2* are often associated with exhaustion, their upregulation during acute activation likely represents a physiological negative feedback mechanism to limit excessive immune responses (39). Combined with the observed upregulation of CD95 (FAS) (**Figure 1D**), this suggests that effector T cells, while executing cytotoxic functions, simultaneously initiate programs conferring sensitivity to Activation-Induced Cell Death (AICD) (40). The co-expression of the anti-apoptotic molecule *BCL2* implies that a subset of cells may engage survival signals, potentially reserving capacity for transition into memory T cells (41) to maintain homeostasis during the contraction phase.

Our motif and footprint analyses further revealed stage-specific involvement of TF families. Early activation (24 h) was dominated by ETS family TFs (e.g., Fli1, ETS1), whereas bZIP family members (e.g., AP-1, BATF) became prominent at 72 h post-stimulation (**Figures 3A–C**). These findings align with previous studies highlighting the critical roles of these TFs in regulating T cell immunity during infection and cancer. For example, Fli1 restrains effector T cell differentiation through suppression of effector-associated genes, and genetic ablation of Fli1 enhances immune protection against both infections and cancers (42). In contrast, BATF counteracts the TOX-driven exhaustion program, overexpression of BATF fosters the generation of long-lived, memory-like CAR-T cells with superior tumor control capacity (43). Precision targeting of stage-specific regulators may allow us to further improve the efficacy of adoptive immunotherapies by endowing T cells with long-term persistence and exhaustion resistance.

Unexpectedly, we observed transcriptional upregulation of exhaustion-associated inhibitory receptors (*CTLA4*, *LAG3*, *TIGIT*, *PDCD1*) as early as 72 h post-stimulation despite diminished chromatin accessibility (**Figure 5A**). This finding contrasts with the stable epigenetic activation of IR genes in exhausted T cells in chronic infection and cancer (24, 44). Based on these observations, we propose a two-phase model in the regulation of IR expression: 1) Transient TCR signaling induces IR transcription in the absence of chromatin remodeling, which is likely driven by TFs such as NFAT/AP-1; 2) Persistent TCR stimulation leads epigenetic fixation of IR gene loci in an open state and sustained expression of these genes. The plasticity of IR loci during acute activation revealed here carries critical therapeutic implications: immune-checkpoint inhibitors (ICIs) may exhibit enhanced efficacy when administered prior to epigenetic fixation of exhaustion. Moreover, combination strategies—such as coupling ICIs with epigenetic modifiers (e.g., BET inhibitors or DNA methyltransferase inhibitors)—could be essential to reverse terminal exhaustion (45, 46).

Our study has several limitations that should be acknowledged. First, although the use of anti-CD3/CD28 beads provides a clear and controllable model for TCR activation, it may not fully recapitulate the complex physiological conditions encountered *in vivo*, particularly within the tumor microenvironment. The current model lacks the integration of diverse signals, such as cytokines, metabolites, and direct cell-cell contacts including those with other immune cell, structural cells at sites of maturation, and any signals T cells receive when interacting with tumor cells. Future studies employing co-culture models with tumor cell lines are warranted to examine how these tumor-derived signals further shape early epigenetic and functional responses. Second, regarding temporal resolution, while we characterized transcriptomic and chromatin accessibility landscapes at 24 and 72 h, the absence of earlier timepoints (e.g., 6 or 12 h) limits our ability to pinpoint the precise timing of the initial molecular dynamics post-activation. Incorporating these earlier activation windows in follow-up work will help capture any immediate onset in epigenetic and/or transcriptional changes. Third, the causal relationship between chromatin accessibility changes and transcriptional output remains to be validated through functional assays, such as CRISPR-mediated perturbation of TF binding sites. Finally, future investigations could also explore how these early epigenetic events influence long-term T cell fate decisions, including memory formation or exhaustion.

In summary, our study systematically delineates the dynamic chromatin remodeling and transcriptional landscapes underlying naïve CD8^+^ T cell activation. We have identified key biological processes involving differentially regulated genes, precisely characterized the chromatin accessibility patterns and expression dynamics of functionally important genes, and uncovered the master transcription factors orchestrating this activation process. These comprehensive findings provide novel mechanistic insights that could inform strategies for optimizing adoptive cellular immunotherapy.

## Data Availability

The data presented in the study are deposited in National Center for Biotechnology Information (NCBI), accession numbers PRJNA1252385 and PRJNA1252400.
